# Polymeric Compounds of Lingonberry Waste: Characterization of Antioxidant and Hypolipidemic Polysaccharides and Polyphenol-Polysaccharide Conjugates from *Vaccinium vitis-idaea* Press Cake

**DOI:** 10.3390/foods11182801

**Published:** 2022-09-11

**Authors:** Daniil N. Olennikov, Vladimir V. Chemposov, Nadezhda K. Chirikova

**Affiliations:** 1 Laboratory of Medical and Biological Research, Institute of General and Experimental Biology, Siberian Division, Russian Academy of Science, 6 Sakhyanovoy Street, 670047 Ulan-Ude, Russia; 2 Department of Biology, Institute of Natural Sciences, North-Eastern Federal University, 58 Belinsky Street, 677027 Yakutsk, Russia

**Keywords:** *Vaccinium vitis-idaea*, lingonberry, polysaccharides, polyphenol–polysaccharide conjugates, arabinogalactan, galacturonan, antioxidant activity, hypolipidemic activity

## Abstract

Lingonberry (*Vaccinium vitis-idaea* L.) fruits are important Ericaceous berries to include in a healthy diet of the Northern Hemisphere as a source of bioactive phenolics. The waste generated by the *V. vitis-idaea* processing industry is hard-skinned press cake that can be a potential source of dietary fiber and has not been studied thus far. In this study, water-soluble polysaccharides of *V. vitis-idaea* press cake were isolated, separated, and purified by ion-exchange and size-exclusion chromatography. The results of elemental composition, monosaccharide analysis, ultraviolet–visible and Fourier-transform infrared spectroscopy, molecular weight determination, linkage analysis, and alkaline destruction allowed us to characterize two polyphenol–polysaccharide conjugates (PPC) as neutral arabinogalactans cross-linked with monomeric and dimeric hydroxycinnamate residues with molecular weights of 108 and 157 kDa and two non-esterified galacturonans with molecular weights of 258 and 318 kDa. A combination of in vitro and in vivo assays confirmed that expressed antioxidant activity of PPC was due to phenolic-scavenged free radicals, nitrogen oxide, hydrogen peroxide, and chelate ferrous ions. Additionally, marked hypolipidemic potential of both PPC and acidic polymers bind bile acids, cholesterol, and fat, inhibit pancreatic lipase in the in vitro study, reduce body weight, serum level of cholesterol, triglycerides, low/high-density lipoprotein–cholesterol, and malondialdehyde, and increase the enzymatic activity of superoxide dismutase, glutathione peroxidase, and catalase in the livers of hamsters with a 1% cholesterol diet. Polysaccharides and PPC of *V. vitis-idaea* fruit press cake can be regarded as new antioxidants and hypolipidemic agents that can be potentially used to cure hyperlipidemic metabolic disorders.

## 1. Introduction

The food industry produces more than one billion tons of waste per year, and the largest part is secondary products generated after mechanic procedures of cleaning, peeling, and pressing of fruits, vegetables, and berries [1]. Recycling of agro-food wastes is the focus of scientists who are interested in eco-friendly technologies for waste processing, which is an important problem of modern life [2]. The problem of fruit processing is of interest to many scientists, but insufficient attention is paid to berry industry waste [3]; at best, this type of waste is composted, and at worst, it is simply thrown away [4]. Among the processed berries, the least attention is paid to hard-skinned berries derived from *Vaccinium* and *Ribes* species, which is explained by the impossibility of its transformation into a puree or a homogeneous mass, despite the use of heat treatment methods [5]. 

Lingonberry (*Vaccinium vitis-idaea* L.) is one of the most common evergreen shrubs of the Ericaceous family in the Northern Hemisphere; it grows in green moss dry coniferous and mixed forests in the mountains, where it rises to the subalpine belt, and in the North, where it reaches subarctic light forests [6]. The lingonberry produces hard-skinned fruits that ripen in August–September as red multi-seeded spherical shiny berries, bearing a dried calyx at the top with a sweet and sour taste. After the first frost, they become soft and watery, and they can winter under snow until spring, but in spring, the berries fall off at the slightest touch. The berries are the reason for the human commercial interest in this plant, especially in the Siberian region, which offers a vast amount of land to allow the collection of more than 50,000 tons of possible annual fruit volume [7]. 

The focus of lingonberry processing on an industrial scale involves high-waste production as a juice, syrup, and jam, and the low-waste production as fruit drying and freezing [8]. The main residue of the high-waste production is a berry press cake, which may be more than half of the initial fruit weight, and most of the fruit processing factories are faced with some waste problems. A possible way to use the capacity of the secondary raw material is isolation of valuable compounds and the development of new products [9]. Isolation of phenolic-rich by-products is a good example of agri-food waste utilization for grapes [10], pomegranates [11], bananas [12], olives [13], oranges [14], and apples [15], which are rich in flavonoids, procyanidins, catechins, hydroxycinnamates, and chalcones [16]. The pomace of chokeberry, blueberry [17], strawberry [18], blackberry [19], and currants [20] is a good source of phenolic antioxidants and dietary fibers that can be used as bioactive products and food additives [21]. In addition to phenolics, agro-industrial fruit waste is a source of polymeric compounds as polysaccharides and polyphenol–polysaccharide conjugates with antioxidant, immunomodulatory, antiobesity and other bioactivities [22].

The phenolics of lingonberry pomace were previously characterized when anthocyanins, catechins, flavonols, and phenolic acids showed antioxidant activity in ABTS^•+^ scavenging capacity and ORAC assays [23], and the extract of lingonberry pomace showed anti-inflammatory properties by inhibition of the NF-κB pathway in LPS-stimulated THP-1 monocytes and COX-2 activity [24]. In the context of deep processing of lingonberry pomace, of special interest is the finding that unprocessed lingonberry pomace has hypolipidemic effects in in vitro assays [21], which may be explained by the high level of dietary fibers or their specific structure. The pomaces of other *Vaccinium* (e.g., blueberries and cranberries) showed in vitro binding of bile acids, which is caused, according to the authors, by the high fiber content [25]. Although there is considerable chemical information about metabolites of *V. vitis-idaea* for which flavonoids, catechins, procyanidins, simple phenolics, and anthocyanins are prevalent [26,27,28], to date, there are no scientific data on lingonberry fibers or polysaccharides. Only subspecies *V. vitis-idaea* ssp. *minus* was studied and showed the presence of neutral and acidic polysaccharides in the water-soluble fraction of polymers with antioxidant and α-glucosidase inhibitory activity [29]. Polysaccharides of other berries, such as goji (*Lycium barbarum*) [30], highland blackberry (*Rubus adenotrichos*) [31], and Cherokee rose (*Rosa laevigata*) [32] have proven their hypolipidemic potential; thus, the wastes of lingonberry processing industries might be a possible source of bioactive polymeric compounds.

As part of the ongoing study of Siberian lingonberry [27,28], the polymeric compounds (polysaccharides and polyphenol–polysaccharide conjugates) from *V. vitis-idaea* press cake produced after juice pressing were isolated, purified, characterized, and its antioxidant and hypolipidemic potential was studied in in vitro and in vivo experiments.

## 2. Materials and Methods

### 2.1. Plant Material and Chemicals

Samples of *V. vitis-idaea* press cake obtained after the juice pressing (moisture content of 88%) were purchased from Yagody Yakutii, Ltd. (Yakutsk, Russia). The plant material was dried in the ventilated heat oven at 40 °C within 7–10 days and stored at 3–4 °C before extraction. The reference standards were purchased from Sigma-Aldrich (St. Louis, MO, USA): polysaccharides-pectin from citrus peel (galacturonic acid ≥ 74.0%; No. P9135), arabinogalactan from larch wood (No. 10830), starch from potato (No. 03967), and microcrystalline cellulose (No. Y0002021); hydroxycinnamates—ferulic acid (≥99%; No. 128708) and sinapic acid (≥98%; No. D7927); bioactivity standards—Trolox (≥97%; No. 238313), cholestyramine (No. 1133004), and simvastatin (≥97%; No. S6196).

### 2.2. Polysaccharide Fraction of V. vitis-idaea Press Cake (VVPS) Isolation

**Figure 1:**
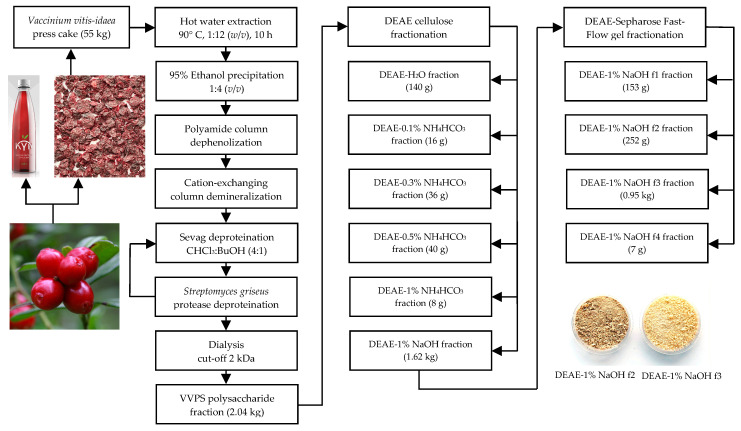
Flow chart for the extraction and fractionation of polysaccharides from *Vaccinium vitis-idaea* press cake. Abbreviation used: VVPS, *V. vitis-idaea* total polysaccharide fraction; DEAE, diethylaminoethyl cellulose; DEAE–sepharose fast-flow gel, diethylaminoethyl cellulose sepharose fast-flow gel.

Dry and milled *V. vitis-idaea* press cake (55 kg) was extracted by hot water (90 °C; press cake:water ratio of 1:12; 10 h) in an industrial extractor (1000 L) coupled with an agitator, filtration unit, vacuum evaporator, condenser, and DNT-1.0 automatic control unit (SBN-Impex, Moscow, Russia) (Figure 1).

The water extract was filtered through industrial filter CUNO™ Self Cleaning Metal Edge Filter Cartridge (200 μm; 3M Purification Inc., Meriden, CT, USA) and concentrated 20 times *in vacuo*, and the residue was mixed with 95% ethanol (1:4). The mixture was left for 24 h, and the crude precipitate was filtered through the cellulose membrane followed by drying, at 50 °C. The yield of crude precipitate was 3.2 kg (5.8% of press cake dry weight). Dry crude precipitate (3 kg) was suspended in 60 L of distilled water, the temperature of the mixture maintained at 60 °C for 1 h, and the resulting solution was filtered through two sequential columns with polyamide (10 kg; Sigma-Aldrich, cat. No. 02395) and a cation-exchanging column (KU-2-8, H^+^-form; Eco-Vita, St. Petersburg, Russia; 20 kg) eluted with 100 L of distilled water (dephenolization and demineralization step). The water eluate was reduced in vacuum, at 30 °C, to 3 L, and the residue was vigorously mixed with a chloroform-*n*-butanol mixture (4:1) in the ratio of 1:1 and centrifuged (6000 rpm, 30 min) (Sevag deproteination step 1). Organic solvents were removed under vacuum, and the water residue was incubated with protease from *Streptomyces griseus* (type XIV, ≥3.5 units/mg; 1 unit per 1 mL of polysaccharide solution; Sigma-Aldrich, cat. No. P5147), at 35 °C, for 3 days (pronase deproteination step 2), and the Sevag deproteination step was repeated. The final solution was dialyzed in benzoylated dialysis tubes (cut-off of 2 kDa; Sigma-Aldrich, cat. No. D2272) against distilled water (48 h), and the non-dialyzed residue was freeze-dried to give *V. vitis-idaea* total polysaccharide fraction (VVPS) as a light-brownish powder in a yield of 2.04 kg (3.7% of press cake dry weight).

### 2.3. Diethylaminoethyl–Cellulose (DEAE–Cellulose) Fractionation of VVPS

The solution of VVPS (2 kg) in water (40 L) was passed through a DEAE–cellulose 52 column (10 kg; Sisco Research Laboratories Pvt. Ltd., New Delhi, India) in HCO_3_^−^ form and eluted with water, NH_4_HCO_3_ solution (0.1%, 0.3%, 0.5%, 1%), and 1% NaOH until there was a negative reaction with phenol–sulfuric acid reagent. All eluates were dialyzed in benzoylated dialysis tubes (cut-off of 2 kDa; Sigma-Aldrich, cat. No. D2272) against distilled water (48 h), and non-dialyzed residues were freeze-dried. The yields of the DEAE–cellulose fractions were 140 g (DEAE–H_2_O), 16 g (DEAE–0.1% NH_4_HCO_3_), 36 g (DEAE–0.3% NH_4_HCO_3_), 40 g (DEAE–0.5% NH_4_HCO_3_), 8 g (DEAE–1% NH_4_HCO_3_), and 1.62 kg (DEAE–1% NaOH).

### 2.4. DEAE–Sepharose Fast-Flow Gel Fractionation of the DEAE–1% NaOH Fraction

The solution of DEAE–1% NaOH (1.6 kg) in water (30 L) was passed through a DEAE–sepharose fast-flow gel column (2 L; GE Healthcare, Chicago, IL, USA) and eluted with water and NaCl solution (0.3%, 0.5%, 0.7%, 0.9%, 1.5%) for detecting the elution progress spectrophotometrically at 190 and 270 nm. All eluates were dialyzed in benzoylated dialysis tubes (cut-off of 2 kDa; Sigma-Aldrich, cat. No. D2272) against distilled water (48 h), and non-dialyzed residues were freeze-dried. The yields of the DEAE–sepharose fast-flow gel fractions were 153 g (DEAE–1% NaOH-f1; elution 0.5% NaCl), 252 g (DEAE–1% NaOH-f2; elution 0.7% NaCl), 0.95 kg (DEAE–1% NaOH-f3; elution 0.9% NaCl), and 7 g (DEAE–1% NaOH-f4; elution 1.5% NaCl).

### 2.5. Chemical Composition of VVPS and DEAE–Cellulose Fractions

Ready-to-use kits for spectrophotometric assays were applied to measure the total carbohydrate content (High Sensitivity Carbohydrate Assay Kit, BioVision, Inc., Milpitas, CA, USA; cat. No. K2049-100), uronic acids (D-Glucuronic/D-Galacturonic Acid Assay Kit, Megazyme, Bray, Ireland; cat. No. K-URONIC), starch (Total Starch Assay Kit, Megazyme; cat. No. K-TSTA-100A), protein (Pierce™ BCA Protein Assay Kit, Thermo Fisher Scientific, Waltham, MA, USA), and phenolics (Phenolic Compounds Assay Kit, Sigma-Aldrich; cat. MAK365). Arabinogalactan–protein complex content was estimated colorimetrically using Yariv reagent as described by Lamport et al. [33], and ash content was determined by the AOAC Official Method^SM^ 942.05 using muffle furnace ignition at 600 °C [34]. All analyses were performed five times, and the data were expressed as the mean value ± standard deviation (S.D.).

### 2.6. Elemental Composition

A 2400 Series II elemental analyzer (Perkin Elmer, Waltham, MA, USA) was used for analysis of carbon, hydrogen, oxygen, and nitrogen contents in the polysaccharides.

### 2.7. Monosaccharide Composition

The monosaccharide composition of polysaccharides was studied after trifluoroacetic acid (TFA) hydrolysis, followed by 1-phenyl-3-methyl-5-pyrazolone (PMP) labeling and HPLC with ultraviolet detection separation (HPLC–UV) as previously described with modifications [35]. Polysaccharide samples (10 mg) were subjected to the hydrolysis procedure with 1 mL of 2 M TFA using sealed ampoules incubated, at 120 °C, for 2 h. After cooling, the mixture was centrifuged (6000× *g*, 15 min) and evaporated in vacuo to remove TFA, and the residue was dissolved in 1 mL of distilled water. The hydrolyzed samples (60 μL) were mixed with 25 μL of 1.5 M NaOH (in water) and 80 μL of 0.5 M PMP (in methanol), incubated at 70 °C (2 h), cooled, and neutralized with 70 μL of 0.5 M HCl. The samples were purified by adding double chloroform (1 mL), followed by vigorous agitation (30 s) and centrifugation (3000× *g*, 10 min). Finally, the organic phase was removed, and the aqueous layer was analyzed by HPLC–UV. HPLC–UV separation of PMP-labeled sugars was performed using a MiLiChrom A-02 microcolumn chromatograph (Econova, Novosibirsk, Russia) coupled with a UV detector and microcolumn ProntoSIL-120-5-C18 AQ (75 mm × 1 mm × 1 μm; Metrohm AG, Herisau, Switzerland) eluted in gradient mode. A solution of 100 mM CH_3_COONH_4_ (pH 6.9) was eluent A, acetonitrile was eluent B, and the following gradient program was used: 0–20 min for 20–26% B. The parameters of column temperature, injection volume, and flow rate were 35 °C, 1 μL, and 150 μL/min, respectively. Chromatograms were recorded at 250 nm. Bidistilled water stock solutions (1 mg/mL) of reference monosaccharides of mannose, ribose, rhamnose, glucose, galactose, xylose, arabinose, fucose, galacturonic acid, and glucuronic acid were prepared and PMP-labeled in the same manner before analysis (Figure S1). The calibration curves were created by plotting the peak area vs. the concentration levels. All analyses were performed in triplicate.

### 2.8. Ultraviolet–Visible (UV–Vis) Spectroscopy

The UV–Vis spectra were obtained using a SF-2000 UV–Vis spectrophotometer (OKB Spectr, St. Petersburg, Russia) in the spectral range of 190–1000 nm using a quartz cell (10 mm). Polysaccharide solutions (5 mg/mL) were prepared using bidistilled water.

### 2.9. Fourier-Transform Infrared (FTIR) Spectroscopy 

An FT-801 Fourier-transform infrared spectrometer (Simex, Novosibirsk, Russia; frequency 4000–600 cm^−1^) with 200 scans and 2-cm^−1^ resolution was used to acquire FTIR spectra. Tablets with potassium bromide (spectroscopic grade) and sample in the ratio of 100:1 were produced by a GS15011 hydraulic press (Specac Ltd., Orpington, UK).

### 2.10. Molecular Weight Determination

A gel permeation–high performance liquid chromatography (GP–HPLC) procedure was used for the molecular weight determination. Experiments were performed on an LCMS 8050 liquid chromatograph coupled with a photodiode array detector (Shimadzu, Columbia, MD, USA) using a Shim-pack Diol-150 column (250 mm × 7.9 mm × 5 μm; Shimadzu) at a column temperature of 25 °C. The eluent was a 10 mM phosphate-buffered solution (pH 7.0). The injection volume was 1 μL, and the elution flow was 1 mL/min. Isocratic elution was applied, and chromatograms were integrated at 190 nm. A series of dextrans (10–410 kDa; Sigma-Aldrich) were used to create a calibration curve. The polysaccharide sample was dissolved in 10 mM phosphate-buffered solution (pH 7.0), centrifuged (6000× *g*), and filtered through a 0.22-μm PTFE syringe filter before injection into the HPLC system for analysis. All analyses were performed in duplicate.

### 2.11. Linkage Analysis 

For linkage analysis, 10 mg of the polysaccharide was methylated by methyl iodide, followed by hydrolysis of the permethylated product using 90% formic acid and 2 M TFA, NaBH_4_ reduction, and acetylation with acetic anhydride [36]. Partially methylated alditol acetates were analyzed by gas chromatography–mass spectrometry using a 5973N gas chromatograph mass spectrometer (Agilent Technologies, Santa-Clara, CA, USA) equipped with a 6890N mass selective detector, a diffusion pump, and an HP-Innowax capillary column (Agilent Technologies; 30 m × 250 μm × 0.50 μm) within a programmed temperature range of 150 to 250 °C, at a rate of 2 °C/min, with helium as the carrier gas (flow rate of 1 mL/min) [37]. The temperature of the transfer line and ion source was 280 °C. The sample injection volume was 1 μL with a split ratio of 50:1, and the scanning range was *m*/*z* 30–400. All analyses were performed in triplicate.

### 2.12. Alkaline Destruction and Analysis of Degradation Products by High-Performance Liquid Chromatography with Photodiode Array Detection and Electrospray Ionization Triple Quadrupole Mass Spectrometric Detection (HPLC–PDA–ESI–tQMS)

Alkaline hydrolysis of the polysaccharides was performed as previously described using 10% potassium hydroxide solution heating, 80% H_2_SO_4_ neutralization, and liquid–liquid extraction with ethyl acetate [38]. Degradation products were analyzed by high-performance liquid chromatography with photodiode array detection and electrospray ionization triple quadrupole mass spectrometric detection (HPLC–PDA–ESI–tQMS) using an LC-20 Prominence liquid chromatograph coupled with an SPD-M30A photodiode array detector (wavelength range 200–600 nm), and an LCMS 8050 triple quadrupole mass spectrometer (Shimadzu, Columbia, MD, USA) with two-eluent gradient elution [39]. The management of the LC–MS system was realized by LabSolution workstation software equipped with an internal LC–MS library. The final identification of metabolites was performed after an integrated analysis of retention time, ultraviolet spectra, and mass spectra in comparison with reference standards and literature data. The relative content of phenolic acids was calculated using calibration curves created using reference substances (ferulic acid, sinapic acid), methanolic solution (1–100 µg/mL) analysis, and by building concentration–peak area graphs. Contents of diferulic and triferulic acids were calculated as ferulic acid equivalents, and disinapic acid was measured as sinapic acid equivalents. All quantitative analyses were performed five times, and the data were expressed as the mean value ± standard deviation (S.D.).

### 2.13. Hydrolysis of DEAE–1% NaOH-f1 and DEAE–1% NaOH-f2 by 2% Oxalic Acid

For mild hydrolysis of polymers DEAE–1% NaOH-f1 and DEAE–1% NaOH-f2, a 500-mg sample was heated with 2% oxalic acid (100 mL), at 100 °C, for 1 h, and the hydrolysate was dialyzed in benzoylated dialysis tubes (cut-off of 2 kDa) against distilled water (48 h), and non-dialyzed residues were freeze-dried. The yields of degraded polymers were DEAE–1% NaOH-f1-d at 130.5 mg and DEAE–1% NaOH-f2-d at 146.5 mg. 

### 2.14. Antioxidant Activity

In vitro microplate spectrophotometric assays of antioxidant activity were used to study the potential of samples to scavenge 2,2-diphenyl-1-picrylhydrazyl radicals (DPPH^•^) [40], 2,2′-azino-bis(3-ethylbenzothiazoline-6-sulfonic acid) cation radicals (ABTS^+•^) [35], superoxide radicals (O_2_^•−^) [39], hydroxyl radicals (OH^•^) [39], and chlorine radicals (Cl^•^) [41], as well as nitric (II) oxide scavenging [42], hydrogen peroxide (H_2_O_2_) inactivating [43], and ferrous ion (Fe^2+^) chelating activity [44]. All analyses were performed five times, and the data were expressed as the mean ± S.D.

### 2.15. Hypolipidemic Activity 

#### 2.15.1. In Vitro Assays

The cholesterol binding capacity of polysaccharides was measured by the method of Nagaoka et al. [45] and an enzymatic Amplex™ red cholesterol assay kit (Thermo Fisher Scientific, Waltham, MA, USA; No. A12216). The bile acid binding properties were studied using the Kim and White assay [46] with an enzymatic bile acid assay kit (Sigma-Aldrich; No. MAK309). The fat binding capacity of polysaccharides was estimated gravimetrically using the Jin et al. assay [47], and pancreatic lipase inhibition was studied spectrophotometrically using *p*-nitrophenol palmitate as a substrate [48]. All analyses were performed three times, and the data were expressed as the mean ± S.D.

#### 2.15.2. In Vivo Assays

The experimental hyperlipidemia was reproduced using the recommendations of Cheng et al. [49] with modification. Fifty male Golden Syrian hamsters (weight of 70–72 g; BioNursery Stezar, Vladimir, Russia) were housed one per cage with a 12 h light/dark cycle (humidity of 50–55%), and regular rodent cholesterol-free chow (Asortiment-Agro Company, Sergiev Posad, Russia) and free access to food and water were provided. After two weeks of adaptation, the animals were weighed and divided into five groups (*n* = 10): (1) the normal diet group; (2) the group with a 1% cholesterol supplementation diet; (3) the group with a 1% cholesterol supplementation diet + simvastatin; (4) the group with a 1% cholesterol supplementation diet + DEAE–1% NaOH-f2 polysaccharide; (5) the group with a 1% cholesterol supplementation diet + with 1% cholesterol diet + DEAE–1% NaOH-f3 polysaccharide. The diets of all groups were switched to a high-fat diet except for the normal diet group over a 3-month period. The general composition of the high-fat diet was 10% lard, 10% yoke powder, 1% cholesterol, and 79% regular rodent cholesterol-free chow. For the next six months, the animals were orally supplemented with simvastatin (10 mg/kg/day; group 3), DEAE–1% NaOH-f2 polysaccharide (250 mg/kg/day; group 4), and DEAE–1% NaOH-f3 polysaccharide (250 mg/kg/day; group 5). Animals in groups 1 and 2 received 0.9% NaCl solution. The experimental procedure was authorized by the Institute of General and Experimental Biology’s Ethical Committee (protocol No. LM-0324, 27 January 2012) before starting the study and was conducted under the internationally accepted principles for laboratory animal use and care. Serum concentrations of total cholesterol, triglycerides, high-density lipoprotein–cholesterol, and low-density lipoprotein–cholesterol were measured enzymatically using Sigma-Aldrich cholesterol assay kit (No. MAK436), serum triglyceride determination kit (No. TR0100), and HDL and LDL/VLDL quantitation kit (No. MAK045), and the malondialdehyde level was measured using a malondialdehyde colorimetric assay kit (Elabscience Biotechnology, Inc., Houston, TX, USA; No E-BC-K025-S). Liver superoxide dismutase, glutathione peroxidase, and catalase were determined using Sigma-Aldrich SOD assay kit (No. 19160), glutathione peroxidase assay kit (No. MAK437), and catalase assay kit (No. MAK100). All analyses were performed five times, and the data were expressed as mean values ± S.D.

### 2.16. Statistical and Multivariate Analysis

Statistical analyses were performed by one-way analysis of variance, and the significance of the mean difference was determined by Duncan’s multiple range test. Differences at *p* < 0.05 were considered statistically significant. The results are presented as the mean ± S.D. The linear regression analysis and generation of calibration graphs were conducted using Advanced Grapher 2.2 (Alentum Software, Inc., Ramat-Gan, Israel).

## 3. Results and Discussion

### 3.1. Yield and Chemical Composition of V. vitis-idaea Press Cake Polysaccharides (VVPS) and DEAE–Cellulose Fractions

Hot water extraction is able to isolate polysaccharides from the press cake *V. vitis-idaea*, which is also typical for other vacciniums such as blueberry, cranberry [25], and Manitoba lingonberry [29]. The total yield of *V. vitis-idaea* polysaccharide fraction (VVPS) after hot water extraction, ethanol precipitation, polyamide column dephenolization, cation-exchange demineralization, two steps of deproteination, and dialysis was 3.7% of dry press cake weight (Table 1). The total carbohydrate level in VVPS was measured to be 92.89%, which included uronic acids at 37.62% and starch at 2.04%. Non-carbohydrate constituents were proteins at 1.67%, phenolics at 2.96%, and ash at 1.56%. The positive test with Yariv reagent implied the presence of arabinogalactan–protein complexes (AGP) estimated as 0.29%. The early study of *Vaccinium* berry polysaccharide, indicating the presence of water-soluble polymers in *V. ashei*, yielded 2.7% of dry berry weight and contained 37% of uronic acids [50]. The purified water-soluble complex polysaccharide from the Northern manitoba lingonberry had 2.1% yield and showed 36% of total carbohydrates, 4.7% of proteins, and 3.5% of phenolics [29].

DEAE–cellulose separation of VVPS resulted in the preparation of six polymer fractions eluted sequentially with water, ammonium bicarbonate (0.1–1%), and sodium hydroxide (1%) (Table 1). The neutral fraction DEAE–H_2_O that eluted first with water showed 7% yield (of VVPS weight), high starch content (25.36%), the highest content of proteins (3.85%), and AGP (3.44%) and no phenolics. 

The subsequent elution with NH_4_HCO_3_ resulted in isolation of four minor fractions with yields of 0.4–2.0%, and NaOH elution gave the dominant fraction DEAE–1% NaOH with 81% yield. Non-water eluted fractions (DEAE-0.1% NH_4_HCO_3_ → DEAE–1% NaOH) showed higher contents of uronic acids (0% → 39.67%), phenolics (0% → 3.54%), and ash (0% → 1.90%) and reduced contents of the starch (9.27% → 0%) and AGP (0.86% → 0%). Polysaccharide fractions of *V. vitis-idaea* press cake obtained through DEAE–cellulose separation were of various yields and chemical compositions.

### 3.2. Elemental Composition

The basic elements of polysaccharides are carbon, hydrogen, and oxygen, and the general formula is C_x_H_y_O_z_. Fraction VVPS showed contents of C, H, and O at 39.08%, 6.14%, 54.52%, respectively, which is typical for carbohydrate polymers, and the composition of the DEAE–cellulose fractions varied in the ranges of 38.94–39.96% (C), 6.10–6.67% (H), and 52.81–54.57% (O) (Table 2).

**Figure 2:**
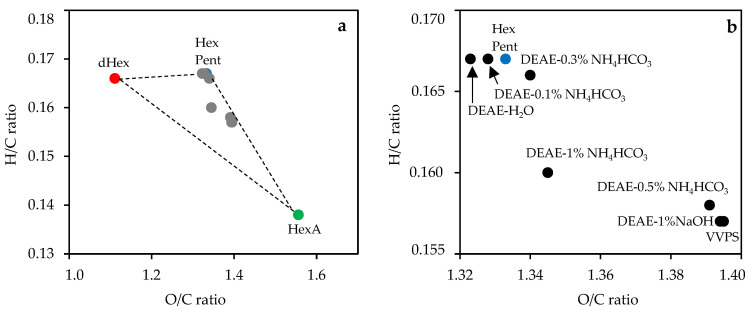
Van Krevelen diagram (correlation between O/C ratio and H/C ratio) for VVPS, DEAE–cellulose fractions (labeled as eluent type), hexose (Hex), pentose (Pent), desoxyhexose (dHex), and hexuronic acid (HexA) (**a**) and its enlarged fragment (**b**). Abbreviation used: VVPS, *V. vitis-idaea* total polysaccharide fraction; DEAE, diethylaminoethyl cellulose.

Nitrogen content was at a zero level (DEAE–0.5% NH_4_HCO_3_, DEAE–0.5% NH_4_HCO_3_), low (DEAE–0.3% NH_4_HCO_3_, DEAE–1% NaOH), or varied from 0.26% (VVPS) to 0.62% (DEAE–H_2_O), which agreed with the previous chemical composition data. To better visualize the elemental composition results, we used a Van Krevelen diagram [51] to plot the atomic O/C ratio as a function of atomic H/C ratio (Figure 2).

Plant polysaccharides traditionally include hexoses, pentoses, hexuronic acids, and desoxyhexoses as structural blocks that are located in the Van Krevelen diagram in different places, creating a “monosaccharide triangle” reflecting extreme points of composition for the possible carbohydrate polymer. Because the monosaccharide composition of the polysaccharides varied in a wide range, the elemental composition data also varied. As shown, VVPS and DEAE–cellulose fractions were inside the triangle, whereas the DEAE–cellulose fraction eluted with water, 0.1%, and 0.3% NH_4_HCO_3_ were closer to the “neutral angle” of hexose (pentose), demonstrating low acidic monosaccharide content. The VVPS and DEAE–cellulose fractions eluted with 0.5% NH_4_HCO_3_ and 1% NaOH were in a lower position, reflecting the presence of uronic acids. The DEAE–cellulose fraction eluted with 1% NH_4_HCO_3_ was in the middle position away from the triangle side Hexose(Pentose)–Hexuronic Acid, which may be due to an increased content of desoxyhexoses.

### 3.3. Monosaccharide Composition

Monosaccharides found in the total polysaccharide fraction of VVPS were neutral glucose, arabinose, and galactose in the ratio of 1.84:1.78:1.00 (58.2 mol% in sum) and minor rhamnose, fucose, xylose, and ribose, as well as acidic galacturonic acid (35.4 mol%) and glucuronic acid (0.6 mol%) (Table 3). The early data on *Vaccinium* polysaccharides showed that the mountain cranberry fruit (*V. vitis-idaea* subsp. *minus*) polysaccharides contain four basic neutral components of arabinose, glucose, galactose, and xylose in the ratio of 6.34:1.73:1.01:1.00 (58.5% in sum) and uronic acids at 39.1% [29]. Caucasian whortleberry fruit (*V. arctostaphylos*) polysaccharides were rich in arabinose (39.2 mol%), galactose (21.0 mol%), and xylose (13.7 mol%) with 18.3% uronic acids [52]. The fruits of rabbiteye blueberry (*V. ashei*) accumulate galacturonic acid in polysaccharides (37.3 mol%) and to a lesser degree arabinose (29.7 mol%), glucose (15.5 mol%), and galactose (10.9 mol%) [50]. Glucose (41.0 mol%) and xylose (33.0 mol%) were the main monosaccharides of European blueberries (*V. myrtillus*) [53], whereas arabinose (36.4 mol%), glucose (25.7 mol%), and galactose (24.5 mol%) dominated in bog blueberries (*V. uliginosum*) [54]. Probably, arabinose, galactose, glucose, and galacturonic acid are essential monosaccharides of *Vaccinium* polysaccharides.

DEAE–cellulose separation of VVPS resulted in isolation of polysaccharide fractions of various monosaccharide compositions (Table 3). Two neutral fractions of DEAE–H_2_O and DEAE–0.1% NH_4_HCO_3_ were composed mainly of glucose, arabinose, and galactose in ratios of 2.02:1.30:1.00 and 1.49:0.92:1.00, respectively.

The subsequent fraction of DEAE–0.3% NH_4_HCO_3_ included 2.8 mol% of galacturonic acid and a reduced level of glucose (28.3 mol%). The decreasing of neutral monosaccharides and occurrence of rhamnose were detected in the DEAE–0.5% NH_4_HCO_3_ fraction with 29.4 mol% of uronic acids. The highest content of rhamnose was found in fraction DEAE–1% NH_4_HCO_3_ (17.6 mol%), and the most acidic fraction was DEAE–1% NaOH with 37.8 mol% of galacturonic acid. These findings were in good correlation with the elemental composition data and demonstrated the fine ability of sequential elution on the DEAE–cellulose to separate the polysaccharide mixture.

### 3.4. Bioactivity of VVPS and DEAE–Cellulose Fractions of V. vitis-idaea Press Cake

Polysaccharides of various *Vaccinium* species are known antioxidants with a potency to inactivate free radicals [52], chelate ferrous ions [50], and bind bile acids [25], making them good antioxidative and lipid-lowering agents. The primary cause of antioxidant potential for plant polysaccharides is phenolic constituents covalently bonded to carbohydrate chains [55], while uronic aids are capable of binding metal ions, bile acids, and cholesterol [56], providing the acidic polysaccharides with bioactivity. Given the diverse chemical properties of *V. vitis-idaea* press cake polysaccharides, we assumed potency for both VVPS and DEAE–cellulose fractions due to the high level of phenolics and uronic acids. 

#### 3.4.1. Antioxidant Activity

Eight in vitro assays were used to study the antioxidant properties of VVPS and DEAE–cellulose fractions against the following free radicals: 2,2-diphenyl-1-picrylhydrazyl radicals (DPPH^•^), 2,2′-azino-bis(3-ethylbenzothiazoline-6-sulfonic acid) cation radicals (ABTS^+•^), superoxide radicals (O_2_^•−^), hydroxyl radicals (OH^•^), and chlorine radicals (Cl^•^); also, nitric (II) oxide scavenging, hydrogen peroxide (H_2_O_2_) inactivating, and ferrous ion (Fe^2+^) chelating activity were examined (Table 4).

Comparative data of VVPS activity and Trolox used as a reference antioxidant demonstrated the better potential of Trolox in scavenging DPPH^•^, ABTS^+•^, O_2_^•−^, OH^•^, Cl^•^, and H_2_O_2_ inactivation, but the total polysaccharide fraction VVPS was an effective scavenger of NO molecules and chelator of Fe^2+^ ions. Interestingly, three commercially available polysaccharides (i.e., pectin from citrus peel, starch, and arabinogalactan) were inactive in scavenging all free radicals, and only pectin demonstrated at least some activity in scavenging NO, H_2_O_2_ inactivation, and good Fe^2+^ chelation.

Analysis of DEAE–cellulose fractions showed DEAE–H_2_O, DEAE–0.1% NH_4_HCO_3_, and DEAE–0.3% NH_4_HCO_3_ as inactive and DEAE–0.5% NH_4_HCO_3_ and DEAE–1% NH_4_HCO_3_ as poorly active. Only fraction DEAE–1% NaOH showed an antioxidant effect comparable to that of VVPS, which indicated the leading role of DEAE–1% NaOH in VVPS antioxidant activity. This fraction has the highest phenolic content, which may explain its excellent antioxidant activity.

The antioxidant activity of the *Vaccinium* polysaccharide fraction from *V. arctostaphylos* showed a 56% scavenging of DPPH^•^ and 49% scavenging of OH^•^ at a dose of 3 mg/mL [52], and the ABTS^+•^ scavenging potential of the *V. ashei* polysaccharide was 20–200 μmol Trolox eq./g [50]. Other berry polysaccharides also were effective scavengers of DPPH^•^ radicals in *Lycium barbarum* with an IC_50_ of 300–400 μg/mL [57], ABTS^+•^ radicals in sweet cherries (*Prunus avium*) and raspberries (*Rubus idaeus*) with an IC_50_ of 57–438 μmol Trolox eq./g [58], and O_2_^•−^ and OH^•^ radicals in blackcurrant (*Ribes nigrum* L.) with IC_50_ values of 0.4–1.0 mg/mL and 0.2–0.5 mg/mL, respectively [59]. Polysaccharide fractions of *V. vitis-idaea* VVPS and DEAE–1% NaOH are excellent antioxidants that are effective against various damaging factors. 

#### 3.4.2. In Vitro Hypolipidemic Activity

In four in vitro assays, polysaccharide VVPS showed the ability to bind bile acids, fat, and cholesterol and inhibit pancreatic lipase (Table 5). The bile acid binding capacity of the reference standard cholestyramine which is the styrene–divinylbenzene copolymer used as a bile acid sequestrant [60] was 10.29 μmol/100 g. Fraction VVPS showed activity value 5.73 μmol/100 g that was much higher than that of the reference polysaccharides such as microcrystalline cellulose (0.07 μmol/100 g), pectin (0.78 μmol/100 g), starch (0.02 μmol/100 g), and arabinogalactan (0.12 μmol/100 g). DEAE fractions of H_2_O, 0.1% NH_4_HCO_3_, 0.3% NH_4_HCO_3_, and 0.5% NH_4_HCO_3_ showed weak activity, but DEAE–1% NaOH was most active (6.04 μmol/100 g). The fat binding levels of VVPS and of fractions DEAE–1% NH_4_HCO_3_ and DEAE–1% NaOH were the highest at 200.02, 231.02, and 252.37 g/100 g, respectively, which were significantly above the activities of microcrystalline cellulose (92.63 g/100 g) and starch (97.67 g/100 g).

Pectin and arabinogalactan demonstrated good fat binding potential with values of 186.85 and 156.14 g/100 g, respectively, and cholestyramine was inactive. A similar pattern was found for cholesterol binding by polysaccharides. Fractions VVPS and DEAE–1% NaOH were the most active (57.02 and 68.37 mg/g, respectively) but were less intensive binders than cholestyramine (93.11 mg/g). Inhibition of pancreatic lipase was detected only for VVPS and DEAE–1% NaOH polysaccharides that had IC_50_ values of 6.24 and 5.33 mg/mL, respectively, exceeding the activity of cholestyramine (IC_50_ 14.02 mg/mL). Thus, polysaccharide fraction VVPS from *V. vitis-idaea* press cake and its active constituent DEAE–1% NaOH showed good in vitro hypolipidemic potential.

Previously, freeze-dried dietary berries showed in vitro bile acid binding with a value ranging from 0.43 μmol/100 g (cranberries, *Vaccinium macrocarpon*) to 0.73 μmol/100 g (blueberry, *Vaccinium* spp.) owing to the high polysaccharide content (particularly dietary fibers) [25]. The bile acid binding potentials of dietary fruits were from 0.21 μmol/100 g for nectarines (*Prunus persica*) to 0.90 μmol/100 g bananas (*Musa paradisiaca*) [61]. Known polysaccharides with bile acid binding activity were also isolated from *Abelmoschus esculentus* [62], *Kadsura coccinea* [63], and *Laminaria japonica* [64], and in all cases, the presence of uronic acids was determined as a basic principle for effect manifestation [65]. The lipid lowering effect of *Inonotus obliquus* and *Volvariella volvacea* polysaccharides was associated with their fat and cholesterol binding [66,67], providing the hypolipidemic activity of polymers. In addition, the pancreatic lipase inhibition plays an important role in the lipid lowering effect of plant food and polysaccharide-derived products. Pectic polysaccharides with varied molecular weight and methoxylation degree are effective pancreatic lipase inhibitors, which is related to a reduction in the surface of the lipid droplet exposed to the enzyme [68], but neutral polysaccharides are also good inhibitors as in the case of *Dictyophora indusiata* polymers [69]. Summarizing the data, the polysaccharide fraction VVPS of *V. vitis-idaea* press cake and its component DEAE–1% NaOH are possible effective antioxidants and potential hypolipidemic agents that need further separation for the isolation of homogenous polymers and their chemical investigation, followed by an *in vivo* study of bioactivity.

### 3.5. Preparative Chromatography of Fraction DEAE-1% NaOH and Characterization of Homogenous Polymers

**Figure 3:**
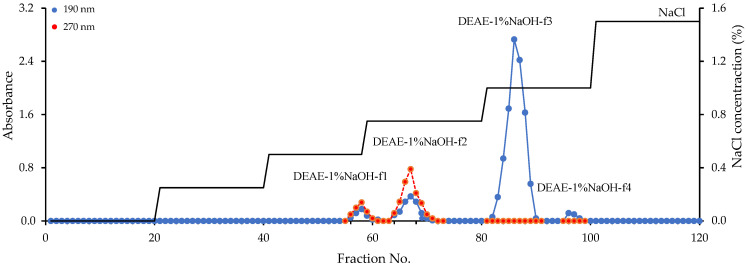
Elution curve of DEAE-1% NaOH fraction on the DEAE–sepharose fast-flow gel.

**Figure 4:**
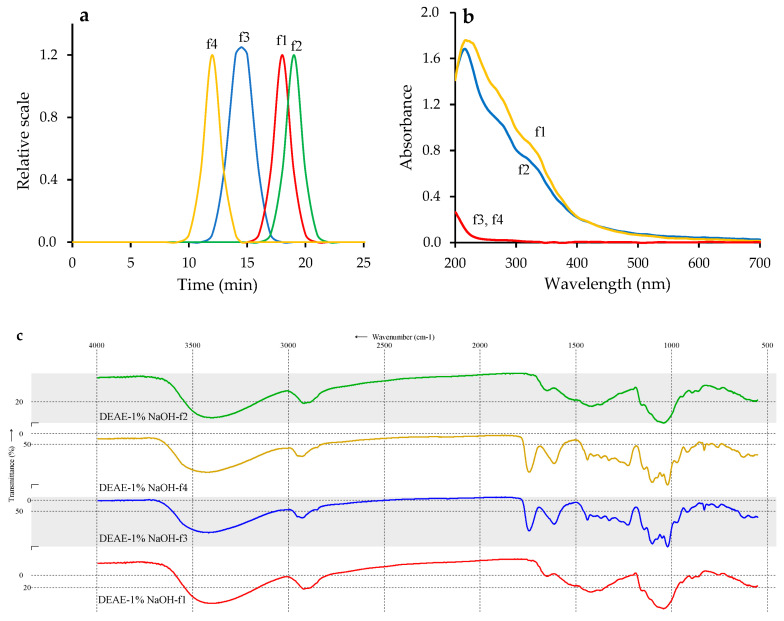
Characteristics of DEAE–sepharose fast-flow gel fractions of DEAE-1% NaOH-f1 (f1), DEAE-1% NaOH-f2 (f2), DEAE-1% NaOH-f3 (f3), and DEAE-1% NaOH-f4 (f4): (**a**) elution profiles on GP-HPLC; (**b**) UV–Vis spectra of 1 mg/mL water solutions; (**c**) FTIR spectra.

The elution curve for the DEAE–sepharose fast-flow gel of the DEAE–1% NaOH fraction demonstrated that it is composed of four polymers labeled as DEAE–1% NaOH-**f1**–DEAE–1% NaOH-f4 (Figure 3). Two polymers, DEAE–1% NaOH-f1 and DEAE–1% NaOH-f2, were UV-positive and detected at 270 nm, unlike DEAE–1% NaOH-f3 and DEAE–1% NaOH-f4, which showed no notable absorption in the UV region. The polymers were isolated, and after re-chromatography, the homogenous fractions were obtained in the yields of 9.5% (DEAE–1% NaOH-f1), 15.6% (DEAE–1% NaOH-f2), 58.4% (DEAE–1% NaOH-f3), and 4.2% (DEAE–1% NaOH-f4) of DEAE–1% NaOH weight. The molecular weights of the polymers were 157.6 kDa (DEAE–1% NaOH-f1), 108.2 kDa (DEAE–1% NaOH-f2), 258.3 kDa (DEAE–1% NaOH-f3), and 318.4 kDa (DEAE–1% NaOH-f4) (Figure 4a, Table 6).

Polymers DEAE–1% NaOH-f1 and -f2 showed strong absorption at 290 ± 3, 310 ± 2 and 330 ± 3 nm caused by the high phenolic content (10.61 and 14.52%, respectively), unlike DEAE–1% NaOH-f3 and -f4, which were weakly absorbed in the UV region (Figure 4b). FTIR spectra of DEAE–1% NaOH-f1 and -f2 were similar, as well as the spectra of DEAE–1% NaOH-f3 and -f4 (Figure 4c). In the spectra of DEAE–1% NaOH-f1 and -f2, intensive bands at 3390 ± 5 and 2920 ± 3 cm^−1^ were attributed to vibrations of O–H and bending vibrations of C–H. Strong bands at 890 ± 2, 1040 ± 3, 1152 ± 2, 1415 ± 2, and 1650 ± 2 cm^−1^ were caused by the vibrations of C–OH, C–O–C, and C–C, and the “fingerprint” region included bands at 850 ± 2 and 910 ± 3 cm^−1^ due to α/β-glycosidic linkages [70]. Specific vibrations in region 1550–1530 cm^−1^ was related to phenolic fragments and phenyl hydroxyl structure [71]. The general profiles of FTIR spectra of DEAE–1% NaOH-f1 and -f2 were very consistent with neutral polysaccharide spectra, e.g., of arabinogalactans and galactans [72].

A more complex spectral pattern was found in the FTIR spectra of DEAE–1% NaOH-f3 and -f4 containing bands typical for the pectic polysaccharides [73]. The low-frequency (<1000 cm^−1^) region of the FTIR spectra included bands of “breathing rings” (762 ± 1 cm^−1^) and α/β-glycosidic linkages (850 ± 2, 890 ± 1, 917 ± 1 cm^−1^). The specific and very intensive “pectic region” (1200–900 cm^−1^) demonstrated vibrations of skeletal C–O and C–C of glycosidic bonds and pyranoid rings at 1025 ± 2, 1050 ± 2, 1075 ± 3, 1097 ± 2, and 1142 ± 2 cm^−1^ [74]. Two intense bans at 1740 ± 3 and 1610 ± 4 cm^−1^ were due to stretching C=O vibrations of esters, and carboxylate anion and symmetric vibrations of COO formed the band at 1415 ± 3 cm^−1^.

Monosaccharide analysis indicated that DEAE–1% NaOH-f1 and -f2 were neutral polymers consisting mainly of arabinose and galactose in ratios of 1.97:1 and 1.51:1, respectively, with minor contents of glucose (0.6–1.7 mol%) and mannose (1.0–1.2 mol%). Polymers DEAE–1% NaOH-f3 and -f4 were characterized by a high content of galacturonic acid (67.8 and 68.0 mol%, respectively), medium levels of arabinose (15.5–16.8 mol%), rhamnose (4.1–6.7 mol%), and galactose (7.1–8.9 mol%), and low contents of mannose (4.1–6.7 mol%) and glucose (2.1–2.7 mol%).

Results of the monosaccharide composition study reflected the arabinogalactan and pectic nature of isolated polymers, as previously confirmed by FTIR and further verified by linkage analysis after methylation and GC-MS analysis. In DEAE–1% NaOH-f1 and -f2, eight peaks were identified as terminal Ara, (1 → 5)Ara, (1 → 3, 5)Ara, terminal Gal, (1 → 3)Gal, (1 → 3, 6)Gal, terminal Glc, and Man. Mild hydrolysis with 2% oxalic acid resulted in formation of degraded linear homopolymers of DEAE–1% NaOH-f1-d (from DEAE–1% NaOH-f1) and DEAE–1% NaOH-f2-d (from DEAE–1% NaOH-f2) consisting only of galactose, which were the core chains of the polymers. This result demonstrated that both neutral polymers were most likely (1 → 3)-linked galactans with branching points located at the *O*-6 positions containing (1 → 3)-linked arabinose chains branched at the *O*-5 positions. The linkage analysis results of DEAE–1% NaOH-f3 and -f4 indicated the dominance of (1 → 4)GalA fragments (60.9–61.3%), which were the building blocks in the construction of the polymer chains. The known data on pectin structure suggest that the rhamnose residues (1 → 2)Rha and (1 → 2, 4)Rha were probably incorporated in the basic polymer chains and that fragments of terminal Ara, (1 → 5)Ara, (1 → 3, 5)Ara, terminal Gal, (1 → 3)Gal, (1 → 3, 6)Gal, terminal Glc, and Man were the blocks for the side chains [75].

**Figure 5:**
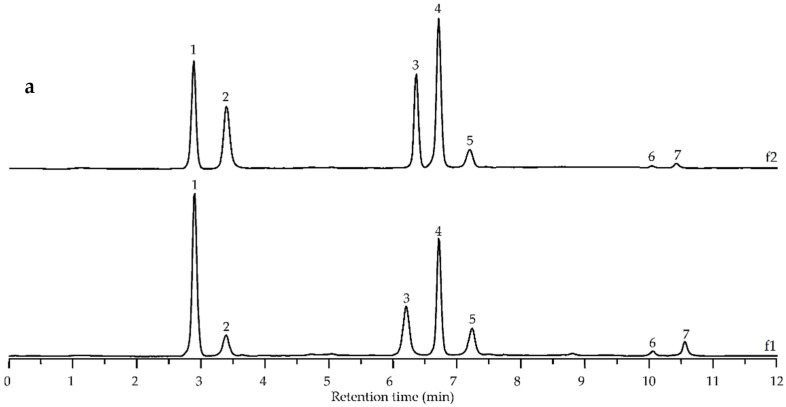

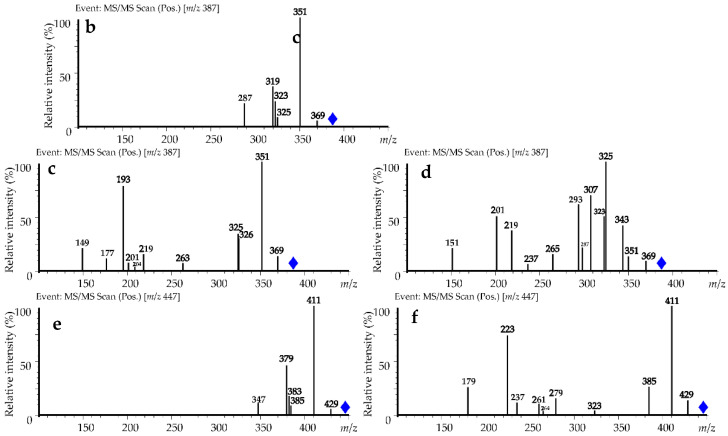
HPLC chromatogram of alkaline destruction products of DEAE–1% NaOH-f1 (f1) and DEAE–1% NaOH-f2 (f2) polymers (**a)** degradation products labeled 1–7 as described in Table 7 and ESI–MS/MS spectra (positive ionization) of degradation products 3 (**b**), 4 (**c**), 5 (**d**), 6 (**e**), and 7 (**f**).

To understand the chemical reasons for DEAE–1% NaOH-f1 and -f2 absorption in the UV spectral region, we degraded both polymers in KOH media followed by HPLC-PDA-MS analysis of the cleavage products. Alkaline hydrolysis of DEAE–1% NaOH-f1 and -f2 resulted in liberation of phenolic acids identified as ferulic acid (*m*/*z* 195; 29.5–38.4%), sinapic acid (*m*/*z* 225; 9.6–15.8%), diferulic acids (*m*/*z* 387; 47.3–51.9%), and disinapic acids (*m*/*z* 447; 2.8–4.7%) (Figure 5, Table 7). Monomeric acids (degradation products 1 and 2) were identified using reference standards, and diferulic and disinapic acids gave a specific combination of daughter ions in the MS/MS spectra, which allowed us to predict their structure. Mass spectral patterns of three diferulic acids 3, 4, and 5 were similar to previously described data of 5-5′-diferulic acid, 8-*O*-4′-diferulic acid, and 8-5′-diferulic acid, respectively (Figure S2) [76]. The spectra of disinapic acids 6 and 7 gave mass spectral patterns that were analogous to those of diferulic acids 3 and 4, but the basic ions were 60 amu larger owing to two methoxy functional groups in the sinapoyl fragments. The predicted structures of 6 and 7 degradation products were 2-2′-disinapic acid and 8-*O*-4′-disinapic acid, respectively (Figure S2). The obtained data showed evidence of polyphenol–polysaccharide conjugates with cross-linked structures of polymers DEAE–1% NaOH-f1 and -f2 esterified by ferulic and sinapic acid and their dimers.

Interestingly, the degradation polymers DEAE–1% NaOH-f1-d and -f2-d formed after elimination of arabinose from DEAE–1% NaOH-f1 and -f2, showed no hydroxycinnamates after alkaline cleavage and likely only occurred if phenolic acids were linked with arabinose residues. This is not unusual because feruloylated arabinose oligomer chains were previously found in the pectin fraction of spinach [77], diferuloyl fragments were found in *Zea mays* cell walls [78,79], and sinapoylated polysaccharides were detected in radish seedlings [80]. Acidic polymers DEAE–1% NaOH-f3 and -f4 released no phenolics after alkaline treatment.

The obtained data showed that homogenic components of the bioactive polysaccharide fraction of *V. vitis-idaea* press cake include four polymers, two of which are polyphenol–polysaccharide conjugates as neutral arabino-3,6-galactans esterified by hydroxycinnamoyl fragments and the other two are pectic-like polysaccharides. Although studies have been conducted on the primary structure of polymers, additional spectral studies need to identify the fine structure of polysaccharides isolated from *V. vitis-idaea*. Looking back at previous information about *Vaccinium* berry polysaccharides, only bilberry (*V. myrtillus*) [53] and rabbiteye blueberry (*V. ashei*) [50] have been mentioned as a source of bioactive polymers of pectic nature without fine structure determination; thus, it is still too early to speculate about genus features in the polysaccharide composition.

### 3.6. Antioxidant and Hypolipidemic Activity of Homogenic Polymers: In Vitro vs. In Vivo Assays

In a series of in vitro antioxidant assays, we found that the polyphenol–polysaccharide conjugates DEAE–1% NaOH-f1 and -f2 were the most active free radical scavengers compared with the parent fraction DEAE–1% NaOH and reference compound Trolox in some cases. The indices of 50% radicals inactivating (IC_50_) were 10.73–12.73 μg/mL against DPPH^•^ (Trolox IC_50_ 8.94 μg/mL), 6.83–7.62 μg/mL against ABTS^+•^ (Trolox IC_50_ 3.25 μg/mL), 70.29–84.75 μg/mL against O_2_^•−^ (Trolox IC_50_ 122.36 μg/mL), and 11.73–14.06 μg/mL against OH^•^ (Trolox IC_50_ 15.23 μg/mL), and the Cl^•^-scavenging potential was 126.79–157.11 mg/g vs. 1000 mg/g for Trolox. The values for NO scavenging and hydrogen peroxide inactivation were 41.09–53.86 μg/mL (Trolox IC_50_ 125.11 μg/mL) and 0.08–0.10 mg/mL (Trolox IC_50_ 0.59 μg/mL), respectively. The acidic polymers DEAE–1% NaOH-f3 and -f4 were inactive in the abovementioned assays. The ability to chelate ferrous ions was revealed for all homogenic polymers but to a greater degree for DEAE–1% NaOH-f1 (8.26 mM Fe^2−^/g) and -f2 (8.72 mM Fe^2−^/g) than for the DEAE–1% NaOH-f3 (6.14 mM Fe^2−^/g) and -f4 (6.09 mM Fe^2−^/g). Polymers with low phenolics content as DEAE–1% NaOH-f3, DEAE–1% NaOH-f3-f4 (Table 4), DEAE–1% NaOH-f1-d, and -f2-d (Table S1) showed low antioxidant potential.

Investigation of the in vitro hypolipidemic activity demonstrated higher bile acid binding potentials for DEAE–1% NaOH-f1 and -f2 polymers of 7.83 and 8.26 μmol/100 g, respectively, vs. 1.85 μmol/100 g for DEAE–1% NaOH-f3 and 1.90 μmol/100 g for DEAE–1% NaOH-f4 (Table 5). Both active polymers were also inhibitors of pancreatic lipase with IC_50_ values of 4.27 and 3.86 mg/mL for DEAE–1% NaOH-f1 and -f2, respectively, whereas DEAE–1% NaOH-f3 and -f4 were inactive. Acidic polysaccharides DEAE–1% NaOH-f3 and -f4 showed fat binding potentials (308.75 and 315.61 g/100 g, respectively), and the cholesterol binding activities of the four homogenic polymers were similar in the range of 59.27–73.92 mg/g. These results mean that the four components of active hypolipidemic fraction DEAE–1% NaOH also have potential to bind bile acids, fat, and cholesterol and inhibit pancreatic lipase, each in its own way. The polyphenol–polysaccharide conjugates showed good bile acid binding and inhibition of pancreatic lipase; however, the acidic non-phenolized polysaccharides were binders of fat, whereas the cholesterol binding activity was at a similar level. The removal of phenolic fragments from DEAE–1% NaOH-f1 and -f2 resulted in significant reduction in the in vitro hypolipidemic activity of the DEAE–1% NaOH-f1-d and -f2-d polymers (Table S2), which indicates the importance of phenolics as active sites of the neutral arabinogalactans.

**Figure 6:**
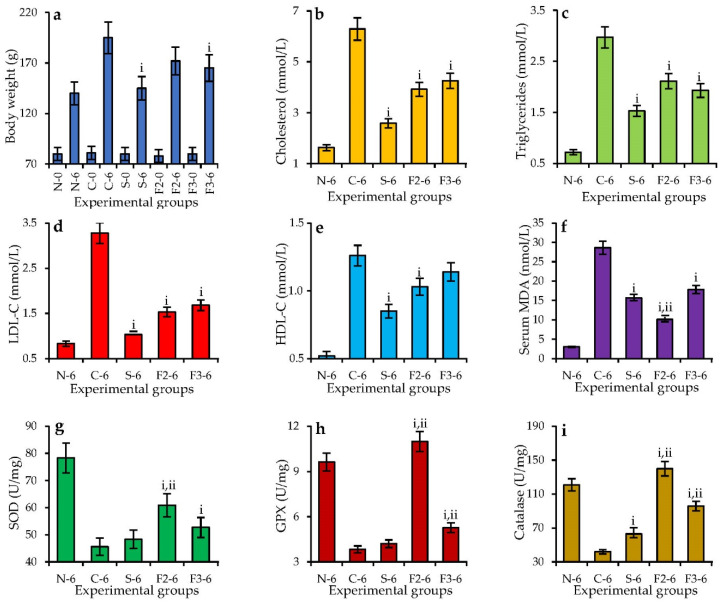
Changes in body weight (**a**), serum total cholesterol (**b**), serum total triglycerides (**c**), serum low-density lipoprotein–cholesterol (LDL–C) (**d**), serum high-density lipoprotein–cholesterol (HDL–C) (**e**), serum malondialdehyde level (MDA) (**f**), liver superoxide dismutase (SOD) (**g**), liver glutathione peroxidase (GPX) (**h**) and liver catalase (**i**) in hamsters with a normal diet (N), with a 1% cholesterol diet (C), with a 1% cholesterol diet + simvastatin (10 mg/kg/day; S), with a 1% cholesterol diet + DEAE–1% NaOH-f2 polysaccharide (250 mg/kg/day; F2), with a 1% cholesterol diet + DEAE–1% NaOH-f3 polysaccharide (250 mg/kg/day; F3) before the feeding period (-0) and after a 6-month feeding period (-6). i—*p* < 0.05 vs. 1% cholesterol diet group (C-group); ii—*p* < 0.05 vs. 1% cholesterol diet + simvastatin group (S-group).

To progress from in vitro to in vivo experiments, we chose polymer DEAE–1% NaOH-f2 as an example of high-yielded polyphenol–polysaccharide conjugates and DEAE–1% NaOH-f3 as high-yielded non-phenolized pectic polysaccharide from *V. vitis-idaea* press cake to treat experimental animals on the standard and high-fat diet (Figure 6). The reference substance was simvastatin, a known hypolipidemic drug, at a dose of 10 mg/kg/day [81]. The high-fat diet with 1% cholesterol resulted in animal body weight gain from 81 ± 4 g at the beginning of the test to a final weight of 195 ± 10 g (vs. 80 ± 4 g → 141 ± 7 g in the standard diet group). The changes in serum lipid profile involved a reduced level of cholesterol (6.29 mmol/L vs. 1.63 mmol/L in the standard diet group), triglycerides (2.97 mmol/L vs. 0.72 mmol/L in the standard diet group), low-density lipoprotein-cholesterol (3.28 mmol/L vs. 0.83 mmol/L in the standard diet group), and high-density lipoprotein–cholesterol (1.26 mmol/L vs. 0.52 mmol/L in the standard diet group). Experimental hyperlipidemia also affected antioxidant status of the animals caused by elevation of the serum malondialdehyde level (28.02 nmol/L vs. 3.02 nmol/L in the standard diet group) and reduction in liver enzymatic antioxidants as superoxide dismutase (SOD; 45.6 U/mg protein vs. 78.3 U/mg protein in the standard diet group), glutathione peroxidase (GPX; 3.83 U/mg protein vs. 9.63 U/mg protein in the standard diet group), and catalase (42 U/mg protein vs. 121 U/mg protein in the standard diet group). This was an indication that the high-fat diet with 1% cholesterol leads to hyperlipidemia associated with antioxidant misbalance. Application of simvastatin lowered the animals’ body weights and reduced serum lipid markers to sustainable levels close to those of the standard diet group, but the antioxidant effect was medium, which was not unexpected and has been previously demonstrated [82]. Both polysaccharides of *V. vitis-idaea* press cake DEAE–1% NaOH-f2 and -f3 demonstrated similar serum lipid lowering effects in the high-fat-diet animals against cholesterol (decreased by 33–38%), triglycerides (decreased by 29–35%), low-density lipoprotein–cholesterol (decreased by 48–53%), and high-density lipoprotein–cholesterol (decreased by 9–18%). This contrasted with the antioxidant potential when polymer DEAE–1% NaOH-f2 was more active than DEAE–1% NaOH-f3. The serum MDA value in the DEAE–1% NaOH-f2 group was 63% lower than in the high-fat diet group, and in the DEAE–1% NaOH-f3 group, we found a 36% reduction. The levels of SOD, GPX, and catalase after application of DEAE–1% NaOH-f2 increased by 60.9%, 186%, and 233%, respectively, compared with those in the high-fat diet group, while DEAE–1% NaOH-f3 resulted in 15%, 37%, and 128% boosts of enzymatic activity, respectively. This means that polysaccharides and polyphenol–polysaccharide conjugates of *V. vitis-idaea* are capable of being antioxidant and hypolipidemic agents in both in vitro and in vivo assays.

Polysaccharides of dietary origin are known hypolipidemic agents that normalize the lipid profile and antioxidant level of high-fat-diet animals [83]. The sources of bioactive polymers are fruits of pumpkin (*Cucurbita pepo*, *C. moschata*) [84,85], Chinese wolfberry (*Lycium barbarum*) [86], jujube or red date (*Ziziphus jujuba*) [87], Cherokee rose (*Rosa laevigata*) [32], and Japanese cornel (*Cornus officinalis*) [88]. In most cases, polysaccharides were polygalacturonates that reduced serum parameters (such as total cholesterol, high/low density lipoprotein cholesterol, and triglycerides) by inhibitory effects on the absorption of the bile acids and cholesterol [89], inhibition of the lipase activity [90], fat and cholesterol binding capacity and reduction in the accumulation of lipids and fecal fat and cholesterol contents [91], modulation of the gene expression of fatty acid synthesis [92], and increased formation of short chain fatty acids in the feces and regulation of lipid metabolism pathways [93]. At the same time, the regulation of observed antioxidant status and improvement of the oxidative stress [55] involves scavenging of free radicals and reduction in liver enzymes [88].

Antioxidant and hypolipidemic potential of polysaccharides and polyphenol–polysaccharide conjugates are markedly linked with structural specifics as molecular weight, elemental and monosaccharide composition, glycosidic linkage, and nature of conjugated polyphenolics [55,83]. The value of phenolic content in carbohydrate polymers is a crucial marker of radical-scavenging ability, nitric oxide (II) and hydrogen peroxide inactivating potential [94], as well as pancreatic lipase inhibition [95]. Additionally, high uronic content is an important factor of metal-chelating activity [96] and binding of bile, fat and cholesterol [83]. The homogenic water-soluble polymers of *V. vitis-idaea* press cake characterized by a high phenolics (polyphenol–polysaccharide conjugates DEAE-1% NaOH-f1 and DEAE-1% NaOH-f2) and uronic content (polysaccharides DEAE-1% NaOH-f3 and DEAE-1% NaOH-f4) and, therefore, the antioxidant and hypolipidemic activity of homogenic polymers, were caused by the various structural factors. Finally, considering the ever-increasing volumes of waste production by lingonberry processing factories, the press cake of *V. vitis-idaea* can become a promising feedstock for bioactive polymers manufacturing.

## 4. Conclusions

This is the first report of lingonberry (*Vaccinium vitis-idaea* L.) fruit press cake polymeric compounds characterization. The results of our study indicate the heterogeneity of *V. vitis-idaea* polysaccharides with a dominance of acidic polymers and polyphenol–polysaccharide conjugates which were neutral arabinogalactans esterified with hydroxycinnamates. This series of in vitro and in vivo studies suggest that polysaccharides normalize the lipid profile and antioxidant status of high-fat-diet hamsters. These findings support the idea of practical use of wastes from food processing as a source for antioxidant and hypolipidemic agent manufacturing in the pharmaceutical industry.

## Figures and Tables

**Table 1:** Yields and chemical composition of VVPS and DEAE–cellulose fractions.

Polysaccharide Fraction	Yield	Total Carbohydrate, % ^a^	Uronic Acids, % ^a^	Starch, % ^a^	Proteins, % ^a^	AGP, % ^a^	Phenolics, % ^a^	Ash, % ^a^
VVPS	3.7 ^b^	92.89 ± 2.78 ^i^	37.62 ± 0.75 ^vii^	2.04 ± 0.04 ^ix^	1.67 ± 0.05 ^xiii^	0.29 ± 0.01 ^xv^	2.96 ± 0.10 ^xix^	1.56 ± 0.03 ^xxii^
DEAE-H_2_O	7.0 ^c^	96.11 ± 2.89 ^ii^	-	25.36 ± 0.52 ^xi^	3.85 ± 0.12 ^xiv^	3.44 ± 0.21 ^xvii^	-	trace
DEAE-0.1% NH_4_HCO_3_	0.8 ^c^	97.14 ± 2.99 ^ii^	-	9.27 ± 0.18 ^x^	1.86 ± 0.05 ^xiii^	0.86 ± 0.03 ^xvi^	-	trace
DEAE-0.3% NH_4_HCO_3_	1.8 ^c^	98.06 ± 3.04 ^iii^	3.22 ± 0.05 ^iv^	0.53 ± 0.01 ^viii^	0.53 ± 0.01 ^xii^	-	-	trace
DEAE-0.5% NH_4_HCO_3_	2.0 ^c^	98.15 ± 3.01 ^iii^	29.53 ± 0.57 ^v^	-	-	-	-	trace
DEAE-1% NH_4_HCO_3_	0.4 ^c^	98.24 ± 3.11 ^iii^	34.12 ± 0.48 ^vi^	-	-	-	0.34 ± 0.01 ^xviii^	0.52 ± 0.01 ^xxi^
DEAE-1% NaOH	81.0 ^c^	92.37 ± 2.76 ^i^	39.67 ± 0.74 ^vii^	-	0.61 ± 0.02 ^xii^	-	3.54 ± 0.12 ^xx^	1.90 ± 0.05 ^xxiii^

^a^*n* = 5. ^b^ Yield—% of dry press cake weight. ^c^ Yield—% of VVPS weight. Values with different numbers (i–xxiii) indicate statistically significant differences among groups at *p* < 0.05 by one-way ANOVA. Abbreviation used: VVPS, *V. vitis-idaea* total polysaccharide fraction; DEAE, diethylaminoethyl cellulose.

**Table 2:** Elemental composition and O/C, H/C ratio of VVPS and DEAE–cellulose fractions, %.

Polysaccharide Fraction	C	H	O	N	O/C	H/C
VVPS	39.08	6.14	54.52	0.26	1.395	0.157
DEAE-H_2_O	39.92	6.65	52.81	0.62	1.323	0.167
DEAE-0.1% NH_4_HCO_3_	39.96	6.67	53.08	0.29	1.328	0.167
DEAE-0.3% NH_4_HCO_3_	39.87	6.62	53.43	0.08	1.340	0.166
DEAE-0.5% NH_4_HCO_3_	39.22	6.21	54.57	-	1.391	0.158
DEAE-1% NH_4_HCO_3_	39.92	6.40	53.68	-	1.345	0.160
DEAE-1% NaOH	38.94	6.10	54.29	0.10	1.394	0.157
Hexose (C_6_H_12_O_6_), pentose (C_5_H_10_O_5_)	40.00	6.67	53.33	-	1.333	0.167
Desoxyhexose (C_6_H_12_O_5_)	43.90	7.31	48.79	-	1.111	0.166
Hexuronic acid (C_6_H_10_O_7_)	37.11	5.15	57.74	-	1.556	0.138

**Table 3:** Monosaccharide composition of VVPS, DEAE–cellulose fractions and *Vaccinium* fruits polysaccharides (PS), mol%.

Polysaccharide Fraction, *Vaccinium* Species	Ara	Gal	Glc	Fuc	Man	Rib	Rha	Xyl	GalA	GlcA
VVPS	22.4	12.6	23.2	0.4	0.9	traces	4.6	traces	35.4	0.6
*V. arctostaphylos* PS [52]	39.2	21.0	6.3	-	-	-	1.5	13.7	18.3 *	
*V. ashei* PS [50]	29.7	10.9	15.5	-	1.2	-	1.6	3.6	37.3	-
*V. myrtillus* PS [53]	4.0	4.0	41.0	-	3.0	-	1.0	33.0	14.0	1.0
*V. uliginosum* PS [54]	36.4	24.5	25.7	-	3.1	-	-	-	10.3	-
*V. vitis-idaea* ssp. *minus* PS [29]	26.8	8.6	14.6	0.3	0.8	-	1.3	8.5	39.1 *	
DEAE-H_2_O	29.3	22.5	45.5	-	2.6	-	-	-	-	-
DEAE-0.1% NH_4_HCO_3_	25.5	27.8	41.4	0.5	4.8	-	-	-	-	-
DEAE-0.3% NH_4_HCO_3_	31.2	33.0	28.3	-	4.7	-	-	-	2.8	-
DEAE-0.5% NH_4_HCO_3_	22.0	29.4	11.5	-	2.9	0.1	4.4	0.3	27.7	1.7
DEAE-1% NH_4_HCO_3_	24.0	19.2	5.0	-	3.6	-	17.6	-	30.6	-
DEAE-1% NaOH	26.2	13.9	0.9	-	0.7	-	3.6	-	54.8	-

* Calculated as a sum of GalA and GlcA.

**Table 4:** Antioxidant activity of VVPS, DEAE–cellulose and DEAE–sepharose fast-flow gel fractions (*n* = 5).

Polysaccharide Fraction	DPPH ^a^	ABTS^+ a^	O_2_^•− a^	OH^• a^	Cl ^b^	NO ^a^	H2O_2_ ^c^	FeCA ^d^
VVPS	35.69 ± 0.73 ^v^	22.59 ± 0.45 ^x^	144.17 ± 4.32 ^xv^	32.60 ± 0.96 ^xix^	27.56 ± 0.55 ^xx^	92.75 ± 2.84 ^xxvii^	0.36 ± 0.01 ^xxxii^	4.71 ± 0.14 ^xxxviii^
DEAE-H_2_O	i.a.	i.a.	i.a.	i.a.	i.a.	i.a.	i.a.	i.a.
DEAE-0.1% NH_4_HCO_3_	i.a.	i.a.	i.a.	i.a.	i.a.	i.a.	i.a.	i.a.
DEAE-0.3% NH_4_HCO_3_	i.a.	i.a.	i.a.	i.a.	i.a.	i.a.	i.a.	i.a.
DEAE-0.5% NH_4_HCO_3_	i.a.	i.a.	i.a.	i.a.	i.a.	i.a.	0.25 ± 0.00 ^xxxi^	0.31 ± 0.01 ^xxxvii^
DEAE-1% NH_4_HCO_3_	i.a.	i.a.	i.a.	i.a.	i.a.	i.a.	0.18 ± 0.00 ^xxxi^	0.24 ± 0.00 ^xxxvi^
DEAE-1% NaOH	24.18 ± 0.48 ^iv^	15.25 ± 0.31 ^ix^	108.26 ± 2.07 ^xiii^	22.86 ± 0.67 ^xviii^	41.67 ± 0.83 ^xxi^	72.11 ± 2.16 ^xxvi^	0.27 ± 0.01	6.83 ± 0.20 ^xxxxi^
DEAE-1% NaOH-f1	12.73 ± 0.25 ^iii^	7.62 ± 0.15 ^viii^	84.75 ± 1.76 ^xii^	14.06 ± 0.42 ^xvii^	126.79 ± 2.53 ^xxii^	53.86 ± 1.61 ^xxv^	0.10 ± 0.00 ^xxx^	8.26 ± 0.34 ^xxxxii^
DEAE-1% NaOH-f2	10.82 ± 0.21 ^ii^	6.83 ± 0.12 ^vii^	70.29 ± 1.70 ^xi^	11.73 ± 0.39 ^xvi^	157.11 ± 3.14 ^xxiii^	41.09 ± 1.20 ^xxiv^	0.08 ± 0.00 ^xxx^	8.72 ± 0.35 ^xxxxii^
DEAE-1% NaOH-f3	i.a.	i.a.	i.a.	i.a.	i.a.	i.a.	i.a.	6.14 ± 0.25 ^xxxx^
DEAE-1% NaOH-f4	i.a.	i.a.	i.a.	i.a.	i.a.	i.a.	i.a.	6.09 ± 0.24 ^xxxx^
Trolox ^e^	8.94 ± 0.18 ^i^	3.25 ± 0.06 ^vi^	122.36 ± 2.44 ^xiv^	15.23 ± 0.36 ^xvii^	1000	125.11 ± 3.75 ^xxviii^	0.59 ± 0.02 ^xxxiii^	0.15 ± 0.00 ^xxxv^
Pectin from citrus peel ^e^	i.a.	i.a.	i.a.	i.a.	i.a.	265.82 ± 10.63 ^xxix^	2.77 ± 0.11 ^xxxiv^	5.26 ± 0.15 ^xxxix^
Starch ^e^	i.a.	i.a.	i.a.	i.a.	i.a.	i.a.	i.a.	i.a.
Arabinogalactan ^e^	i.a.	i.a.	i.a.	i.a.	i.a.	i.a.	i.a.	0.22 ± 0.01 ^xxxvi^

^a^ IC_50_, μg/mL; ^b^ Trolox-equivalents, mg/g; ^c^ IC_50_, mg/mL; ^d^ mM Fe^2+^/g; ^e^ Reference standards. Values with different numbers (i–xxxxii) indicate statistically significant differences among groups at *p* < 0.05 by one-way ANOVA. Abbreviation used: i.a., inactive; VVPS, *V. vitis-idaea* total polysaccharide fraction; DEAE, diethylaminoethyl cellulose.

**Table 5:** In vitro hypolipidemic activity of VVPS, DEAE–cellulose and DEAE–sepharose fast-flow gel fractions (*n* = 5).

Polysaccharide Fraction	Bile Acids Binding, μmole/100 g	Fat Binding, g/100 g	Cholesterol Binding, mg/g	Pancreatic Lipase Inhibition, IC_50_, mg/mL
VVPS	5.73 ± 0.22 ^viii^	200.02 ± 7.24 ^xvi^	57.02 ± 2.56 ^xxiii^	6.24 ± 0.18 ^xxx^
DEAE-H_2_O	0.10 ± 0.00 ^ii^	103.75 ± 3.70 ^xii^	15.37 ± 0.63 ^xx^	i.a.
DEAE-0.1% NH_4_HCO_3_	0.14 ± 0.00 ^ii^	127.80 ± 4.49 ^xiii^	18.62 ± 0.80 ^xxi^	i.a.
DEAE-0.3% NH_4_HCO_3_	0.27 ± 0.01 ^iii^	131.29 ± 4.63 ^xiii^	19.83 ± 0.93 ^xxi^	i.a.
DEAE-0.5% NH_4_HCO_3_	0.92 ± 0.04 ^v^	139.16 ± 4.90 ^xiii^	37.10 ± 1.69 ^xxii^	i.a.
DEAE-1% NH_4_HCO_3_	3.62 ± 0.16 ^vii^	231.02 ± 8.14 ^xvii^	59.22 ± 2.50 ^xxiii^	i.a.
DEAE-1% NaOH	6.04 ± 0.29 ^viii^	252.37 ± 8.97 ^xvii^	68.37 ± 3.02 ^xxiv^	5.33 ± 0.15 ^xix^
DEAE-1% NaOH-f1	7.83 ± 0.39 ^ix^	183.70 ± 6.40 ^xv^	72.11 ± 3.24 ^xxv^	4.27 ± 0.12 ^xxviii^
DEAE-1% NaOH-f2	8.26 ± 0.44 ^ix^	173.11 ± 6.04 ^xv^	73.92 ± 3.36 ^xxv^	3.86 ± 0.10 ^xxvii^
DEAE-1% NaOH-f3	1.85 ± 0.09 ^vi^	308.75 ± 10.83 ^xviii^	59.27 ± 2.65 ^xxiii^	i.a.
DEAE-1% NaOH-f4	1.90 ± 0.10 ^vi^	315.61 ± 10.88 ^xviii^	60.08 ± 2.72 ^xxiii^	i.a.
Cholestyramine *	10.29 ± 0.40 ^x^	i.a.	93.11 ± 4.15 ^xxvi^	14.02 ± 0.42 ^xxxi^
Microcrystalline cellulose *	0.07 ± 0.00 ^ii^	92.63 ± 3.12 ^xi^	10.33 ± 0.45 ^xix^	i.a.
Pectin from citrus peel *	0.78 ± 0.03 ^iv^	186.85 ± 6.51 ^xv^	57.82 ± 2.69 ^xxiii^	i.a.
Starch *	0.02 ± 0.00 ^i^	97.67 ± 3.43 ^xi^	9.63 ± 0.44 ^xix^	i.a.
Arabinogalactan *	0.12 ± 0.00 ^ii^	156.14 ± 5.46 ^xiv^	21.16 ± 0.90 ^xxi^	i.a.

* Reference standards. Values with different numbers (i–xxxi) indicate statistically significant differences among groups at *p* < 0.05 by one-way ANOVA. Abbreviation used: i.a., inactive; VVPS, *V. vitis-idaea* total polysaccharide fraction; DEAE, diethylaminoethyl cellulose.

**Table 6:** Yield, molecular weight, monosaccharide composition, phenol content, and phenolic acid content after alkaline hydrolysis and linkage analysis of DEAE–sepharose fast-flow gel homogenic polymers and degradation polymers.

Parameter	DEAE-1% NaOH-f1	DEAE-1% NaOH-f1-d	DEAE-1% NaOH-f2	DEAE-1% NaOH-f2-d	DEAE-1% NaOH-f3	DEAE-1% NaOH-f4
Yield	9.5 ^a^	26.1 ^b^	15.6 ^a^	29.3 ^c^	58.4 ^a^	4.2 ^a^
*M_w_*, kDa ^d^	157.6 (±1.4%)	35.5 (±1.0%)	108.2 (±1.9%)	25.4 (±1.4%)	258.3 (±2.2%)	318.4 (±2.7%)
*M_w_*/*M_n_* ^d^	1.56 (±2.9%)	1.64 (±3.7%)	1.48 (±2.1%)	1.42 (±2.6%)	1.71 (±3.7%)	1.62 (±2.4%)
Monosaccharide composition, mol%						
Ara	65.1	-	58.6	-	15.5	16.8
Gal	33.1	99.9	38.7	99.9	7.1	8.9
Glc	0.6	-	1.7	-	0.2	0.1
Man	1.2	-	1.0	-	2.7	2.1
Rha	-	-	-	-	6.7	4.1
GalA	-	-	-	-	67.8	68.0
Linkage analysis, molar ratio						
Terminal Ara	12.6	-	12.4	-	3.7	3.2
1,5-Ara	38.4	-	31.6	-	10.3	10.9
1,3,5-Ara	14.1	-	15.0	-	1.2	2.8
Terminal Gal	7.1	1.0	10.2	1.4	8.2	7.9
1,3-Gal	10.5	98.9	9.1	98.5	4.2	5.7
1,3,6-Gal	15.7	-	19.4	-	2.6	3.0
1,4-Gal	-	-	-	-	60.9	61.3
1,2-Rha	-	-	-	-	4.6	3.6
1,2,4-Rha	-	-	-	-	2.0	0.6
Terminal Man	1.0	-	0.4	-	2.0	0.9
Terminal Glc	0.6	-	1.9	-	0.3	0.1
Phenols, % ^e^	10.61 (±0.32)	-	14.52 (±0.44)	-	<0.1	<0.1

^a^ Yield, % of DEAE-1%NaOH DW. ^b^ % of DEAE-1% NaOH-f1 DW. ^c^ % of DEAE-1% NaOH-f2 DW. ^d^ *n* = 3. ^e^ *n* = 5.

**Table 7:** Characterization and content of degradation products released after alkaline destruction of polymers DEAE-1% NaOH-f1 and DEAE-1% NaOH-f2.

Compound (No. Figure 5)	ESI-MS, [M + H]^+^, *m*/*z*	ESI-MS/MS, *m*/*z*	Content after Alkaline Destruction, % ^a^	
			DEAE-1% NaOH-f1	DEAE-1% NaOH-f2
Ferulic acid (1)	195	181	38.4 ± 1.1	29.5 ± 0.8
Sinapic acid (2)	225	211, 197	9.6 ± 0.2	15.8 ± 0.4
Diferulic acid (3)	387	369, 351, 325, 323, 319, 287	10.5 ± 0.1	12.7 ± 0.2
Diferulic acid (4)	387	369, 351, 326, 325, 263, 219, 204, 201, 193, 177, 149	28.6 ± 0.4	35.9 ± 0.7
Diferulic acid (5)	387	369, 351, 343, 325, 323, 307, 297, 293, 265, 237, 219, 201, 151	8.2 ± 0.2	3.3 ± 0.1
Disinapic acid (6)	447	429, 385, 383, 411, 379, 347	1.4 ± 0.0	0.8 ± 0.0
Disinapic acid (7)	447	429, 411, 401, 323, 279, 264, 261, 237, 223, 205, 179, 177	3.3 ± 0.1	2.0 ± 0.0

^a^ Percentage of total peak area. *n* = 3.

## Data Availability

Data is contained within the article.

## Supplementary Materials

The following are available online at https://www.mdpi.com/article/10.3390/foods11182801/s1: Table S1: Antioxidant activity of degradation polymers DEAE-1% NaOH-f1-d and DEAE-1% NaOH-f2-d; Table S2: In vitro hypolipidemic activity of degradation polymers DEAE-1% NaOH-f1-d and DEAE-1% NaOH-f2-d; Figure S1: HPLC-UV chromatograms of PMP-labeled samples of blank, standard monosaccharide mixture, and 2 M TFA hydrolysates of VVPS polysaccharide, fraction VVPS: DEAE-H_2_O, and fraction VVPS: DEAE-1% NAOH; Figure S2: Possible ways of MS/MS cleavage of degradation products 3–7 released after alkaline destruction of DEAE-1% NaOH-f1 and DEAE-1% NaOH-f2 polymers.
