# Supplementary material for: Polymeric Compounds of Lingonberry Waste: Characterization of Antioxidant and Hypolipidemic Polysaccharides and Polyphenol-Polysaccharide Conjugates from Vaccinium vitis-idaea Press Cake
Source: Foods. 2022 Sep 11;11(18):2801. doi: 10.3390/foods11182801 (PMC9497698; doi:10.3390/foods11182801)
Supplement: Supplementary file 1 [file foods-11-02801-s001.zip › foods-1891708-supplementary.pdf]

foods

Supplementary Materials

Polymeric Compounds of Lingonberry Waste: Characterization of Antioxidant and Hypolipidemic Polysaccharides and Polyphenol-Polysaccharide Conjugates from *Vaccinium vitis-idaea* Press Cake

Daniil N. Olennikov [1,*], Vladimir V. Chemposov [2] and Nadezhda K. Chirikova [2]

[1] Laboratory of Medical and Biological Research, Institute of General and Experimental Biology, Siberian Division, Russian Academy of Science, 670047 Ulan-Ude, Russia
[2] Department of Biology, Institute of Natural Sciences, North-Eastern Federal University, 677027 Yakutsk, Russia
* Correspondence: olennikovdn@mail.ru; Tel.: +8-902-160-06-27

Supplementary Content

Table S1. Antioxidant activity of degradation polymers DEAE-1% NaOH-f1-d and DEAE-1% NaOH-f2-d.

Table S2. *In vitro* hypolipidemic activity of degradation polymers DEAE-1% NaOH-f1-d and DEAE-1% NaOH-f2-d.

Figure S1. HPLC-UV chromatograms of PMP-labeled samples of blank, standard monosaccharide mixture, and 2 M TFA hydrolysates of VVPS polysaccharide, fraction VVPS: DEAE-H_2O, and fraction VVPS: DEAE-1% NAOH

Figure S2. Possible ways of MS/MS cleavage of degradation products 3–7 released after alkaline destruction of DEAE-1% NaOH-f1 and DEAE-1% NaOH-f2 polymers.

Table S1. Antioxidant activity of degradation polymers DEAE-1% NaOH-f1-d and DEAE-1% NaOH-f2-d.

Polysaccharide	DPPH•[a]	ABTS+•[a]	$O_2^{\bullet -}$[a]	OH•[a]	Cl•[b]	NO[a]	H_2O_2[c]	FeCA[d]
DEAE-1% NaOH-f1-d	> 100	> 100	> 250	> 100	< 5	> 500	> 5	< 0.01
DEAE-1% NaOH-f2-d	> 100	> 100	> 250	> 100	< 5	> 500	> 5	< 0.01

[a] IC_{50}, μg/mL; [b] Trolox-equivalents, mg/g; [c] IC_{50}, mg/mL; [d] mM Fe^{2+}/g.

Table S2. *In vitro* hypolipidemic activity of degradation polymers DEAE-1% NaOH-f1-d and DEAE-1% NaOH-f2-d.

Polysaccharide fraction	Bile acids binding, μmole/100 g	Fat binding, g/100 g	Cholesterol binding, mg/g	Pancreatic lipase inhibition, IC_{50}, mg/mL
DEAE-1% NaOH-f1-d	0.01 ± 0.00	Inactive (< 1)	2.28 ± 0.11	Inactive (> 30)
DEAE-1% NaOH-f2-d	0.01 ± 0.00	Inactive (< 1)	2.09 ± 0.08	Inactive (> 30)

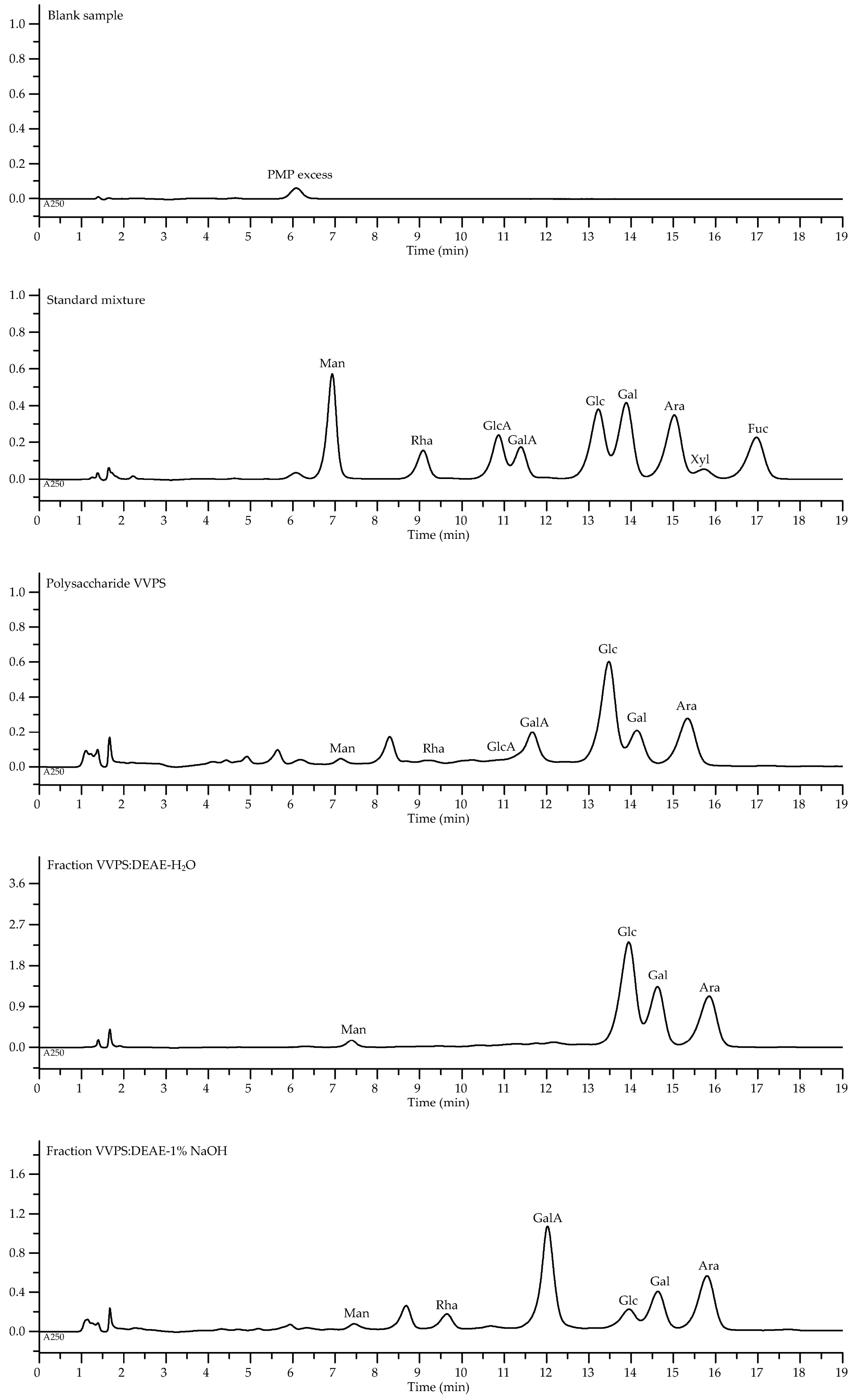

Figure S1. HPLC-UV chromatograms of PMP-labeled samples of blank (**a**), standard monosaccharide mixture (**b**), and 2 M TFA hydrolysates of VVPS polysaccharide (**c**), fraction VVPS: DEAE-H_2O (**d**), and fraction VVPS: DEAE-1% NAOH (**e**). Relative retention times (t_R) of monosaccharides: Man = 1.00; Rha = 1.35; GlcA = 1.59; GalA = 1.68; Glc = 1.94; Gal = 2.04; Ara = 2.21; Xyl = 2.31; Fuc = 2.50.

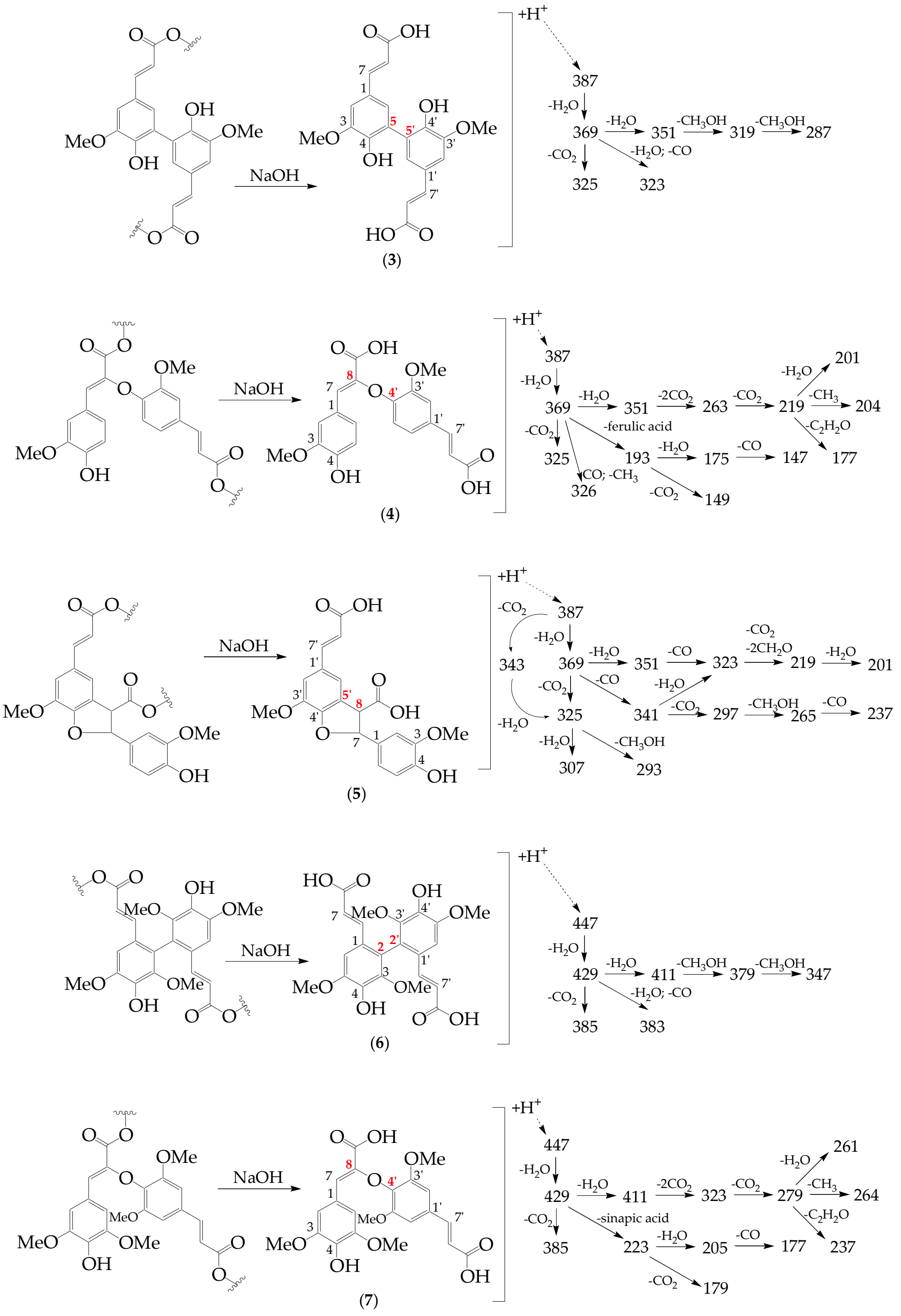

Figure S2. Possible ways of MS/MS cleavage of degradation products 3–7 released after alkaline destruction of DEAE-1% NaOH-f1 and DEAE-1% NaOH-f2 polymers.
